# Assessing the Veterans Health Administration’s response to intimate partner violence among women: protocol for a randomized hybrid type 2 implementation-effectiveness trial

**DOI:** 10.1186/s13012-020-0969-0

**Published:** 2020-05-07

**Authors:** Katherine M. Iverson, Melissa E. Dichter, Kelly Stolzmann, Omonyêlé L. Adjognon, Robert A. Lew, LeAnn E. Bruce, Megan R. Gerber, Galina A. Portnoy, Christopher J. Miller

**Affiliations:** 1 Women’s Health Sciences Division, National Center for PTSD, VA Boston Healthcare System, 150 South Huntington Ave (116B-3), Boston, MA 02130 USA; 2 Department of Psychiatry, Boston University School of Medicine, Boston, MA USA; 3 VA Center for Health Equity Research and Promotion (CHERP), Corporal Michael J. Crescenz VA Medical Center, 3900 Woodland Ave, Philadelphia, 19104 PA USA; 4 Department of Social Work, Temple University, Philadelphia, PA USA; 5 Center for Healthcare Organization and Implementation Research (CHOIR), VA Boston Healthcare System, 150 S. Huntington Ave (152 M), Boston, MA 02130 USA; 6 Massachusetts Veterans Epidemiology Research and Information Center, VA Boston Healthcare System, 150 S. Huntington Ave (152 M), Boston, MA 02130 USA; 7 Intimate Partner Violence Assistance Program, Care Management and Social Work, Department of Veterans Affairs, 810 Vermont Avenue, Washington, DC 20420 USA; 8 Department of Social Work, Western Kentucky University School of Social Work, Bowling Green, KY USA; 9 Women’s Health Center, VA Boston Healthcare System, 150 S. Huntington Ave, Boston, MA 02130 USA; 10 Section of General Internal Medicine, Boston University School of Medicine, Boston, MA USA; 11 Pain, Research, Informatics, Medical comorbidities, and Education (PRIME) Center, VA Conneticut Healthcare System, 950 Campbell Avenue, West Haven, CT 06516 USA; 12 Yale School of Medicine, New Haven, CT USA; 13 Department of Psychiatry, Harvard Medical School, Boston, MA USA

**Keywords:** Intimate partner violence, Women Veterans, Screening, Primary care, Stepped wedge

## Abstract

**Background:**

Intimate partner violence (IPV) against women in the United States (US) remains a complex public health crisis. Women who experience IPV are among the most vulnerable patients seen in primary care. Screening increases the detection of IPV and, when paired with appropriate response interventions, can mitigate the health effects of IPV. The Department of Veterans Affairs (VA) has encouraged evidence-based IPV screening programs since 2014, yet adoption is modest and questions remain regarding the optimal ways to implement these practices, which are not yet available within the majority of VA primary care clinics.

**Methods/design:**

This paper describes the planned evaluation of VA’s nationwide implementation of IPV screening programs in primary care clinics through a randomized implementation-effectiveness hybrid type 2 trial. With the support of our VA operational partners, we propose a stepped wedge design to compare the impact of two implementation strategies of differing intensities (toolkit + implementation as usual vs. toolkit + implementation facilitation) and investigate the clinical effectiveness of IPV screening programs. Using balanced randomization, 16–20 VA Medical Centers will be assigned to receive implementation facilitation in one of three waves, with implementation support lasting 6 months. Implementation facilitation in this effort consists of the coordinated efforts of the two types of facilitators, external and internal. Implementation facilitation is compared to dissemination of a toolkit plus implementation as usual. We propose a mixed methods approach to collect quantitative (clinical records data) and qualitative (key informant interviews) implementation outcomes, as well as quantitative (clinical records data) clinical effectiveness outcomes. We will supplement these data collection methods with provider surveys to assess discrete implementation strategies used before, during, and following implementation facilitation. The integrated-Promoting Action on Research Implementation in Health Services (i-PARIHS) framework will guide the qualitative data collection and analysis. Summative data will be analyzed using the Reach Effectiveness Adoption Implementation Maintenance (RE-AIM) framework.

**Discussion:**

This research will advance national VHA efforts by identifying the practices and strategies useful for enhancing the implementation of IPV screening programs, thereby ultimately improving services for and health of women seen in primary care.

**Trial registration:**

NCT04106193. Registered on 23 September 2019.

Contributions to the literature

- Women who experience intimate partner violence (IPV) are among the most vulnerable patients seen in primary care. IPV screening programs are effective in detecting IPV and connecting women to care, but we need to understand how to systematically and effectively implement IPV screening programs in busy primary care settings.
- A cluster randomized, stepped wedge, hybrid type 2 implementation-effectiveness design will evaluate implementation and clinical effectiveness outcomes.
- A mixed method examination of implementation strategies associated with implementation facilitation and mapping them to the Expert Recommendations for Implementing Change compilation will advance implementation science.

## Background

Intimate partner violence (IPV) against women, defined as psychological, physical, and sexual aggression from a past or current intimate partner, is a complex public health problem. Although men also experience IPV, women are more likely to experience severe violence and to face more physical and mental health-related impacts [1, 2]. Nearly 7 million women are physically assaulted, raped, or stalked by an intimate partner in the USA annually [1], and IPV is strongly associated with poorer physical, psychological, and social health [3, 4]. Physical health problems range from injuries directly caused by physical and sexual assaults, to other chronic nervous system, cardiovascular, and reproductive conditions [5, 6]. Psychological problems include posttraumatic stress disorder, depression, anxiety, substance abuse, and suicidality [5–7]. Women who experience IPV are 2.4 times more likely to attempt suicide than those who do not experience IPV [8]. Social consequences include homelessness, financial insecurity, and unemployment [9–12]. The burden of IPV on women and society underscores the need for a feasible and effective health care response [13, 14].

Women who experience IPV present frequently to primary care [15–17], which is recognized as an ideal setting for safely identifying women who experience IPV and offering them resources and referrals to health and social services [18]. The United States Preventive Services Task Force [18] and recent research have found evidence that routine screening—paired with an appropriate response to disclosure—can reduce IPV and physical and mental health harms in women of childbearing age [19, 20]. One study found that women who talked to a provider about IPV were four times more likely to use an intervention and 2.6 times more likely to exit the relationship [21].

Routine screening for IPV in primary care is important for the US Department of Veterans Affairs (VA) medical centers, as nearly one in five (19%) Women Veterans (WVs) seen in primary care have experienced IPV in the past year [17] and IPV is more prevalent among WVs compared to women who have not served in the military [22]. Screening WVs for IPV is vital because formative research demonstrates that WVs want to be asked and are more likely to disclose when asked directly [23, 24]. Thus, VA recommends an evidence-based, trauma-informed, and patient-centered approach to IPV screening programs, including respect for patient privacy and autonomy, with an emphasis on three components: IPV screening on an annual basis, brief risk assessment and provision of resources for WVs who report experiencing IPV, and psychosocial service referrals (when desired by patients) [25].

A growing number of VA-based women’s specific primary care clinics have established IPV screening practices consistent with these recommendations [26, 27]. Yet, about two thirds of WVs accessing primary care services in VA do so in mixed-gender clinics or those that share space with clinics that predominantly treat men, such clinics have been slower to adopt evidence-based IPV screening practices. As such, there is a current disparity in the access to IPV screening programs for women seen in mixed-gender settings. To meet this need, in 2018, the study investigators launched a partnership with VA’s Office of Women’s Health Services and the IPV Assistance Program of Care Mangement and Social Work Services to evaluate the implementation of IPV screening practices for WVs in mixed-gender (model 1) and shared space (model 2) primary care clinics. In this manuscript, we describe the recently funded randomized program evaluation trial stemming from these efforts.

## Methods

### Overview

We have designed a stepped wedge hybrid type 2 implementation-effectiveness cluster randomized trial [28] to investigate both implementation and clinical effectiveness outcomes associated with the rollout of evidence-based IPV screening programs for WVs in VA-based mixed-gender and shared-space primary care clinics. We have received approval from the VA Boston Healthcare System’s Institutional Review Board (IRB) for all study procedures to evaluate this operations-led effort. The integrated-Promoting Action on Research Implementation in Health Services (i-PARIHS) framework [29] will guide the qualitative data collection and analysis. Summative data will be analyzed using the Reach Effectiveness Adoption Implementation Maintenance (RE-AIM) framework [30].

### Aims

For this study, we will be comparing two distinct implementation strategies to support the uptake of IPV screening programs. First, all sites will receive a toolkit designed by the IPV Assistance Program, to be distributed to local staff responsible for encouraging IPV screening in primary care (toolkit plus implementation as usual [IAU]). Second, consistent with our stepped wedge design, during the study, each site will be assigned (in a staggered fashion) to cross over to more intensive implementation support in the form of implementation facilitation [31]. Implementation facilitation will entail external Office of Women’s Health Services experts working directly with the local primary care staff at participating sites to encourage IPV screening uptake and will also involve toolkit dissemination (toolkit plus implementation facilitation).

An additional aim of the study will be to compare two IPV screening tools. Specifically, a 5-item screener has been validated to detect probable IPV in primary care settings [9, 32], but feedback from the field to Women's Health Services leadership suggests the length of the 5-item screener may represent an implementation barrier in busy practices. Thus, as a secondary aim, we will compare the utility of the 5-item screener to a 1-item screener. Consistent with the RE-AIM Framework, the proposed research has the following specific aims:

1. Estimate the degree of reach, adoption, implementation fidelity, and maintenance achieved using two different implementation strategies (toolkit + IAU vs. toolkit + implementation facilitation) (implementation aim)
2. Evaluate the clinical effectiveness of IPV screening programs, as evidenced by disclosure rates and post-screening psychosocial service use (i.e., social work and mental health services uptake) (clinical effectiveness aim)
  2a. Compare the clinical effectiveness of two IPV screening tools (5-item vs. 1-item screener) in terms of disclosure rates and post-screening psychosocial service use
3. Identify multi-level barriers to and facilitators of IPV screening program implementation and sustainment

### Stepped wedge trial design

**Fig. 1:**
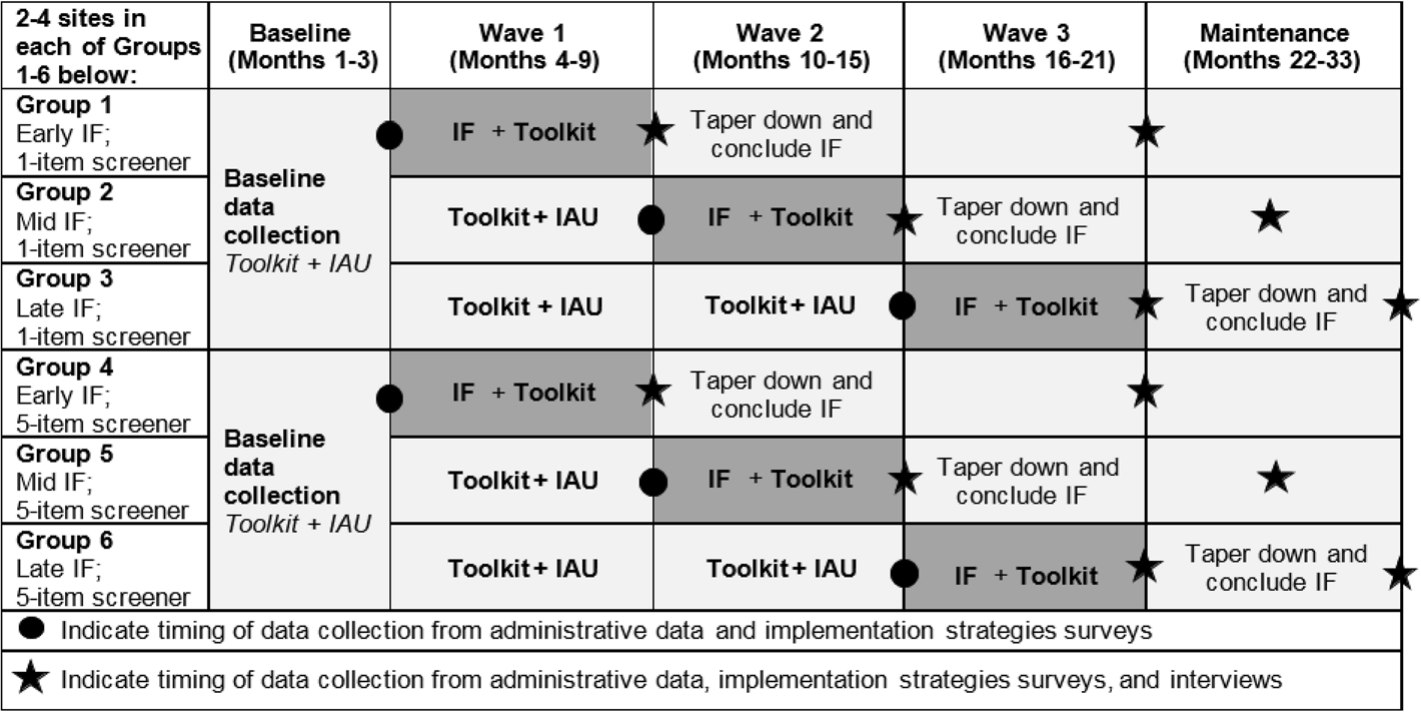
Stepped wedge design and approximate timing of data collection activities. Stepped wedge design (light gray cells denote toolkit + implementation as usual [IAU]; dark gray cells denote implementation facilitation [IF] support)

We will use a stepped wedge controlled trial design [33], such that all sites will start with the less intensive implementation strategies (toolkit + IAU) before receiving the more intensive implementation support (toolkit + implementation facilitation) in a staggered fashion (Fig. 1). Stepped wedge designs have their roots in balanced incomplete block designs [34]. A stepped wedge has the advantage of minimizing burden on implementation support personnel as start times are staggered, with all sites ultimately receiving the implementation facilitation strategy. We aim to recruit 16–20 VA Medical Centers (VAMCs) as sites, with about six sites assigned to each of the three waves with start times staggered by 6-month intervals. All sites will start with 3 months in the less-intensive toolkit + IAU condition to allow the collection of baseline data. When each site switches to the more intensive intervention, they will receive 6 months of active implementation facilitation followed by 6 months of step-down.

Our stepped wedge design is novel due to the inclusion of the two types of sites to each wave. This design will accommodate aim 2a above, in that sites within each wave will be assigned to use either the 5-item or 1-item IPV screener. This structure, however, creates novel challenges in assigning sites to study waves. Specifically, we had previously developed a balancing algorithm to minimize the imbalance on key facility-level characteristics between waves in a stepped wedge while retaining some of the benefits of randomization [35, 36]. For the current study, we incorporated our partners’ input in developing a similar algorithm featuring different facility-level variables of contextual relevance to IPV screening program implementation in primary care (Table 1). However, for the current study, we also need to ensure balance between the sites assigned to the 5-item vs. 1-item screener. Thus, our balancing algorithm will identify the least-imbalanced combinations of site assignments across the three study waves and the two screener types on the variables included in Table 1. We will then randomly select from among the 2% least-imbalanced site assignment combinations.

**Table 1:** Variables in site assignment balancing algorithm

Construct	Operationalization
1) Total number of non-women’s specific primary care clinics	Total number of mixed gender and separate but share space primary care clinics at the facility (i.e., women’s health primary care model 1 and model 2 clinics)
2) Veterans Integrate Service Network (VISN)	VISN in which the facility is located (regional network)
3) Women Veterans treated in primary care	Number of Women Veterans seen within targeted clinics at the facility
4) Primary care workload	Panel size per primary care provider at the facility
5) Primary care functioning related to mental health	Percentage of primary care patients seen for embedded mental health services with Primary Care Mental Health Integration (PCMHI) clinicians
6) Rurality	Binary; coded as “1” if facility is located within rural county or at least 65% of their patients live in rural areas
7) Facility complexity	VA designation based on several facility variables such as size and availability of certain types of specialty services
8) Emergency Department capacity	Number of Emergency Department visits at the facility
9) Reliance of Women Veterans on VA care	Percent of Women Veterans who get prescriptions at the facility
10) Mental health service capacity	Mental health encounters per unique patient at the facility

### Implementation procedures

#### Toolkit + IAU

All participating VAMCs will initially receive a toolkit, developed by the IPV Assistance Program, to support the uptake of IPV screening programs. The toolkit will include the following tools to encourage the adoption and tailoring of IPV screening programs at individual facilities: (1) VHA’s recommendations for IPV screening programs; (2) VHA’s protocol for screening, response, and referrals; (3) templates notes with screening, response, and disposition; (4) resources to guide clinical response following screening (e.g., IPV brochure, risk assessments, and safety planning tool) and community resources; (5) documentation guidance addressing issues of privacy and safety; and (6) training tools (e.g., PowerPoint slides). In addition, as a condition for participation, all sites must designate a local staff person (e.g., member of the women’s primary aligned care team (PACT)) responsible for launching IPV screening for WVs in primary care at that site. This designated local staff person will fill the internal facilitator role once the more intensive toolkit + implementation facilitation condition begins, as described next.

#### Toolkit + implementation facilitation

Based on our stepped wedge design, all participating VAMCs will be assigned to start receiving implementation facilitation support in wave 1, 2, or 3; this implementation facilitation will be tailored to support either the 5-item or 1-item screener as appropriate (Fig. 1). Implementation facilitation is defined as a multifaceted process of enabling and supporting individuals and groups and has been widely used in primary care settings as an umbrella strategy for overcoming barriers and leveraging strengths to foster implementation of evidence-based interventions and care delivery models [31, 37, 38]. It involves the coordinated efforts of the two types of facilitators, external and internal [39]. The core components of implementation facilitation have been specified and will be assessed during the proposed study [39].

External facilitators are located outside of the local VAMC, implementing the innovation and providing high-level implementation expertise and support. External facilitation will be provided by two Office of Women’s Health Services external facilitators who are clinical experts in IPV and have completed an intensive implementation facilitation training through VHA’s Behavioral Health Quality Enhancement Research Initiative (QUERI) Program’s Implementation Facilitation Training Hub. Operational partners in the VHA IPV Assistance Program provide additional consultation to the Office of Women’s Health Services consultants. Internal facilitators are located within the VAMC and provide boots-on-the-ground knowledge to assist with the implementation. These internal facilitators will collaborate closely with the external facilitators (e.g., through regular email correspondence and a minimum of monthly phone meetings) to support the local implementation of the core components of IPV screening program. Internal facilitators are most often expected to be a member of a women’s health Primary Aligned Care Team (i.e., women’s health physician, nurse, and/or medical director) in collaboration with the facility’s IPV Assistance Program Coordinator. Congress recently funded these coordinator positions nationwide, paving the way for these individuals to play a vital role in supporting the primary care clinics in implementing IPV screening programs.

### Quantitative evaluation of the implementation outcomes

We propose to use RE-AIM as an evaluation framework to guide our qualitative analyses [30]. RE-AIM examines 5 dimensions: reach into the target population, effectiveness of the intervention, adoption by the setting, implementation fidelity, and maintenance (i.e., degree of sustainment over time). Of these, one (effectiveness of the intervention) pertains to clinical effectiveness (aim 2), while the remaining four relate to implementation (aims 1 and 3). Thus, RE-AIM is ideal for structuring the proposed hybrid implementation-effectiveness evaluation. Table 2 describes the specific outcome measures and data sources for each RE-AIM dimension. Given our hybrid type 2 design, we are equally interested in implementation and clinical effectiveness outcomes. For this program evaluation, our primary implementation outcome will be reach (i.e., the proportion of WVs eligible for IPV screening who receive the screening). Our primary clinical effectiveness outcome will be disclosure rates and post-screening psychosocial care use (i.e., the proportion of women with a positive IPV screen who accept a referral and use psychosocial services in the ensuing 2 months).

**Table 2:** RE-AIM guides evaluation of the impact and clinical effectiveness of IPV screening programs

RE-AIM dimension (study aim)	Outcome measures	Numerator	Denominator	Data sources
Reach (aim 1): The proportion of eligible WVs receiving IPV screening program.	Proportion of WVs seen in primary care clinics during the last 3 months of each study phase who receive IPV screening	WVs who receive IPV screening as indicated by documentation (templated note, clinical reminder)	WVs seen in primary care clinics who are eligible for IPV screening (WVs seen in clinic with no screen in prior year)	VA Corporate Data Warehouse (CDW): IPV screening status from health factors in templated notes and clinical reminders
Effectiveness (aim 2): The clinical effectiveness of IPV screening programs on disclosure and post-screening psychosocial service use.	a. Proportion of eligible WVs who screen positive for IPV	a. WVs who screen positive for IPV	a. WVs who were screened for IPV	CDW: IPV screening status, responses, and referral disposition (accept or decline referrals) from health factors in note templates and clinical reminders; psychosocial service visits based on clinic stop codes
	b. Proportion of WVs accepting psychosocial service referrals who use such services within 2 months	b. WVs who accepted referral and who use ≥ 1 psychosocial services within 2 months after positive screen	b. WVs who screen positive for IPV and accept a referral	
Adoption (aim 1): The absolute number/proportion of primary care clinics using the IPV screening program.	a. Proportion of primary care clinics completing IPV screening with at least 70% of eligible WVs during evaluation periods	a. Primary care clinics completing IPV screening with at least 70% of eligible WVs during evaluation periods	a. All primary care clinics who saw at least one WV eligible for IPV screening during evaluation periods	CDW: WV patient encounters within primary care clinics. IPV screening status from health factors in note templates and clinical reminders. IPV screening status, resource provision, and referrals offered determined by checkboxes as health factors in note templates and clinical reminders
	b. Proportion of primary care clinics delivering IPV screening program to at least 70% of eligible WVs during evaluation periods, including evidence of resource provision and referral offered for those with positive screens	b. Primary care clinics delivering the IPV screening program to at least 70% of eligible WVs during evaluation periods, including evidence of resource provision and referral offered for those with positive screens	b. All primary care clinics who saw at least one WV eligible for IPV screening during evaluation periods	
Implementation fidelity (aim 1): The extent to which IPV screening programs are conducted as intended by clinics.	Proportion of clinics for whom at least 60% of WVs accepting referrals attend psychosocial services within 2 months of positive screen Note: Additionally assessed via key informant interviews	Primary care clinics for whom at least 60% of WVs accepting referral attend 1 + psychosocial visits within the ensuing 2 months	All primary care clinics who referred at least one screen-positive WV to psychosocial services	CDW: IPV screening status, responses, and referral disposition (accept or decline referrals) from health factors in templated notes and clinical reminders; psychosocial service visits based on clinic stop codes
Maintenance (aims 1 and 3): The degree to which IPV screening programs are sustained over time.	Repeat Reach analysis in last 3 months of study. Note: Implementation strategies and contextual factors impacting maintenance will be assessed via survey and key informant interviews	Same as Reach analysis above, but with data collection occurring 9–12 months after IF ends	Same as Reach analysis above, but with data collection occurring 9–12 months after IF ends	CDW: For repeat of Reach analysis

We will extract relevant administrative and clinical data for all VAMCs from the VA Corporate Data Warehouse (CDW) for all five dimensions of RE-AIM (see Table 1 below for details). The templated note or clinical reminder is required for the study and includes health factors for IPV screening (i.e., disclosure), response (i.e., resources provided), and referrals offered (i.e., accepted or declined IPV Assistance Program-Coordinator referral; accepted or declined psychosocial services referral). Psychosocial care use, and time to first post-screening psychosocial care use, also come from CDW based on clinic stop codes for social work, psychology, psychiatry, primary care mental health integration, drug or alcohol treatment, and housing services.

Our operation partners are interested in testing whether a brief 1-item screener can be as effective as a more comprehensive (but lengthier) 5-item screener a modified Hurt, Insult, Threaten, Scream tool [40], which has been validated for use with the WV population [9, 32]. The tool asks individuals to indicate how often in the past year a current or past partner has done any of the following: “insulted or talked down to you,” “screamed or cursed at you,” “threatened you with harm,” “physically hurt you,” or “pressured or forced you to have sexual activities.” Response options for each item ranges from 1 (never) to 5 (frequently). Endorsement of any item indicates a positive screen. A more efficient screening tool could potentially enhance the uptake of IPV screening programs. However, prior to formal adoption of a 1-item tool, it is critical to evaluate the effectiveness of the tool in eliciting IPV disclosures (sub-aim 2a). The item was developed by a panel of IPV and women’s health clinical experts and researchers and consists of “In the past 12 months, have you experienced insulting, screaming, threatening, hitting, or unwanted sexual activity by a former or current partner?”

We will use repeated measures generalized estimating equations (GEE) [41–43] analyses to address our study aims while controlling for site characteristics (between-site effects) and calendar time, which reflects secular trends (within-site effects). GEE quantifies and apportions the variance in outcomes among relevant factors, thus isolating the change in outcome due to the primary contrast of interest. It extends the traditional general linear model with a continuous outcome (e.g., linear regression) to accommodate binary outcomes for each subject and count outcomes for each site. GEE also accommodates repeated measures (within-subject correlation), random effects (subject), moderate imbalance among independent factors (sites), and various types of missing data. We will include relevant demographic and clinical variables at the patient level (e.g., age, mental health diagnoses, recent psychosocial service use) as covariates. Furthermore, GEE will allow us to explore the results for the patterns of unequal variance, relevant correlation structures, and variance component models to ensure that our results are robust. Our planned sample size will also support the exploration of many site-specific effects by adding site-interaction terms to the model. This method will also allow us to explore the study outcomes using a nested approach (WVs within VAMCs within study conditions). We will use the same GEE-based approach for encompassing each of the five RE-AIM outcome domains included in Table 2 above. Specifically, each primary outcome listed in Table 2 can be expressed as a proportion, and so columns for numerators and denominators are included. Analyses in each of these domains within the GEE framework will consist of logistic regression with random effects.

We will repeat these analyses to compare the clinical effectiveness of the 5-item tool and 1-item tool as well. To evaluate the extent to which IPV screening programs facilitate timely access to psychosocial services, we compare the median number of days to first new psychosocial service visits following a positive screen for women who accepted referrals during and after the IF + toolkit implementation period. In sum, our GEE analyses will allow us to determine the extent to which changes in the quantitative outcome measures described in Table 2 prior to the implementation facilitation differ along two dimensions: (1) based on the changes during and after the implementation period and (2) based on the use of the 5-item vs. 1-item screener.

We will supplement these quantitative data, derived from the CDW, with a 50-item electronic survey to all internal facilitators at pre-facilitation, post-facilitation, and the sustainment phases to assess the use of discrete implementation strategies at each site during each study period. The survey assesses 50 of the 73 strategies described by the Expert Recommendations for Implementing Change (ERIC) study [44], which has shown validity when strategies are assessed via survey [45]. To reduce participant burden, only the 50 strategies most germane to the IPV screening program implementation are queried (i.e., excluded financial incentives as this is irrelevant in VA) and we also inquire about the perceived effectiveness of each strategy on a Likert-type scale. The responses will inform a deeper dive into the use and perceived effectiveness of implementation strategies in subsequent internal facilitator interviews (see Qualitative evaluation of the implementation outcomes and adaptations made to IPV screening programs section).

### Power analyses

Given our hybrid type 2 design, we conducted power analyses for implementation outcomes (aim 1) and clinical effectiveness outcomes (aim 2). For aim 1, we specifically estimated power related to the RE-AIM dimension of reach (the proportion of WVs receiving the IPV screening under each of the two implementation conditions, toolkit + IAU vs. IF + toolkit). Based on a previous research examining VA healthcare use among women screened for IPV [46, 47] and our estimated sample size, we are powered above 90% to detect expected differences between conditions, allowing a type I error rate of 5%. For aim 2, we are also powered above 90% to detect expected differences in post-screening psychosocial visit, again allowing a type I error rate of 5%.

### Qualitative evaluation of the implementation outcomes and adaptations made to IPV screening programs

To contextualize our RE-AIM findings and address aim 3, we will conduct interviews with 2–3 key informants at each site including clinicians, administrators, and internal facilitators. Key informant interviews are an ideal method for the proposed research as they elicit in-depth information from individuals with first-hand knowledge of the factors influencing local IPV screening programs [48]. The interviews will be guided by a semi-structured interview guide, with open-ended questions and prompts to elicit organic feedback. Interviews will occur following the toolkit + implementation facilitation phase, and 1 year later to assess sustainment. We will base our qualitative analyses on the integrated-Promoting Action on Research Implementation in Health Services framework, a determinant implementation framework that will enable us to characterize and explain the ways in which IPV screening programs have and have not been successfully implemented and sustained (aim 3 [29]). Consistent with the core integrated-Promoting Action on Research Implementation in Health Services constructs, our interview guide and qualitative analyses will assess the impact of factors specific to the local clinical context, IPV screening procedures, implementation facilitation, and the recipients of the innovation on the success of the implementation effort, as done in our formative research on IPV screening programs [26]. We expect that collaboration both within the primary care clinic (e.g., nurses, PCPs, and social workers) and with other services, particularly social work and mental health, will be relevant recipients.

Furthermore, guided by the Wiltsey-Stirman framework [49], we will use our qualitative interview results to assess the adaptations made to IPV screening programs. Identifying those adaptations, and their impacts on IPV screening, will allow us to determine and make recommendations to our partners to facilitate the range of adaptations that are acceptable and avoid those that are not. The qualitative interviews will include additional probes for the use of core implementation facilitation activities and the use of implementation strategies defined by the Expert Recommendations for Implementing Change project [44]. This will inform the time and skills needed to facilitate the implementation of IPV screening programs by our operation partners at other VAMCs.

Rapid content analysis [50, 51] using a hybrid inductive-deductive approach will efficiently reveal IPV screening program practices, adaptations, implementation strategies, and multi-level barriers to and facilitators of implementation and sustainability of IPV screening programs. We will transfer interview and site summaries into matrices and use matrix analysis methods to examine our key domains of (1) IPV screening, response, and referral practices; (2) adaptations; (3) implementation strategies by time, including core implementation facilitation activities and Expert Recommendations for Implementing Change defined strategies; (4) toolkit engagement; (5) barriers to and facilitators of IPV screening program implementation; and (6) barriers to and facilitators of IPV screening program maintenance. Matrices systematically note the similarities, differences, and trends in responses across sites, thereby expediting synthesis and summary of findings [52]. We will use a hybrid deductive and inductive analytic approach [50, 51], where prior constructs and assumptions are evaluated against the data and new themes are incorporated into the coding scheme [53]. For aim 3, we will characterize barriers to and facilitators of implementation and sustainability within and across VAMCs per the RE-AIM domain of maintenance (Table 2). Other sources of quantitative data (e.g., survey responses) will be triangulated with the matrix analysis to provide additional context for findings.

### Advisory board

We will convene an advisory board of operations partners and key stakeholders in WV’s health care, IPV Assistance Program, primary care program implementation, implementation science, and WVs. The use of an advisory board is an established community-based participatory research strategy that will help frame and monitor the progress of the study while providing guidance on values and practices to enhance the feasibility and acceptability of future implementation efforts [54].

### Limitations and anticipated challenges

A limitation of the proposal is the lack of information about patients’ experiences with the IPV screening program. For example, some patient-level factors that might affect willingness to be screened for IPV or accept psychosocial referrals and engage in follow-up psychosocial services. To address this issue, we have ensured that WVs are represented on our Advisory Board. In addition, our analyses of service use are limited by the reliance on clinical reminder/note templates and the types of psychosocial services accessed in VA. Currently, it is not possible to ascertain whether WVs who experienced IPV access community services (e.g., the National Domestic Violence Hotline). This type of information will be queried generally during key informant interviews, but examining community partnerships and care coordination for IPV is a step for future research. We also recognize that unforeseeable circumstances experienced by our operational partners or participants (i.e., turnover) may impact the execution of the rollout; we have overpowered the study for both of our primary study aims to ensure that our results will be robust even if our original recruitment or site participation goals are not met. Finally, despite its advantages, our use of a nested stepped wedge design—technically a quasi-experimental design—has certain limitations: it precludes subject-level randomization, introduces possible time trends, and means that we do not have a true control group as would be the case in a traditional parallel groups randomized controlled trial. However, our use of a balancing algorithm should minimize time trends, and our GEE analytic approach will allow us to identify such trends. Furthermore, a traditional randomized controlled trial was not appropriate from the perspective of our clinical partners and was not practical from a resource management perspective.

## Discussion

There is an urgent need to better support women who are experiencing IPV. VA-based primary care clinics are an ideal setting to implement evidence-based IPV screening programs that can lead to the provision of appropriate healthcare services and other resources for WV’s who may be experiencing IPV. The study protocol described in this manuscript—a stepped wedge hybrid II implementation-effectiveness trial—was developed in close partnership with relevant operations partners in VA and will use state-of-the-art evaluation methods to answer key questions regarding how best to implement and sustain such IPV screening programs. Specifically, our mixed quantitative and qualitative data collection will allow us to develop clear guidance for our operations partners regarding context-sensitive implementation strategies to address multi-level barriers to program implementation and sustainment. Finally, we will make recommendations to help facilitate acceptable adaptations in clinical practices and avoid those that are not. This will help ensure the effectiveness and efficiency of future and ongoing efforts to address IPV.

## Data Availability

Not applicable, as this manuscript does not contain any data.

## Abbreviations

CDW: Corporate Data Warehouse
GEE: Generalized estimating equations
HSR&D: Health Services Research and Development
IAU: Implementation as usual
IF: Implementation facilitation
IPV: Intimate partner violence
RE-AIM: Reach Effectiveness Adoption Implementation Maintenance
SDR: Service Directed Research
VA: US Department of Veteran Affairs
VAMCs: VA Medical Centers
VHA: Veterans Health Administration
WVs: Women Veterans
